# The New Invincibles: HIV Screening among Older Adults in the U.S

**DOI:** 10.1371/journal.pone.0043618

**Published:** 2012-08-27

**Authors:** Oluwatoyosi A. Adekeye, Harry J. Heiman, Onyekachi S. Onyeabor, Hyacinth I. Hyacinth

**Affiliations:** 1 National Center for Primary Care, Morehouse School of Medicine, Atlanta, Georgia, United States of America; 2 Satcher Health Leadership Institute, Morehouse School of Medicine, Atlanta, Georgia, United States of America; 3 Department of Microbiology, Biochemistry and Immunology, Morehouse School of Medicine, Atlanta, Georgia, United States of America; Tulane University, United States of America

## Abstract

**Background:**

Thirteen percent of the U.S. population is ages 65 and older, a number projected to reach 20% by 2030. By 2015, 50% of Human Immunodeficiency Virus (HIV)-infected individuals in the U.S. are expected to be ages 50 and older. Current Centers for Disease Control and Prevention guidelines recommend “opt-out” HIV screening for individuals ages 13–64. The purpose of this study was to assess the occurrence and barriers to HIV screening in older adults, and to evaluate the rationale for expanding routine HIV screening to this population.

**Methods:**

The study used 2009 National Health Interview Survey (NHIS) data. A total of 12,366 (unweighted) adults, ages 50 and older, participated in the adult section of the NHIS and answered questions on the HIV/AIDS, Sexually Transmitted Diseases, and Tuberculosis components. Associations between HIV screening, socio-demographic variables, and knowledge of HIV-related disease were examined using logistic regression models.

**Results:**

The HIV screening rate within this population was 25.4%. Race had no statistically significant effect. Low risk perception of HIV exposure (84.1%) accounted for low likelihood of planned screening (3.5%) within 12 months post survey. A routine medical check-up was the single most common reason for HIV screening (37.6%), with only about half (52.7%) of the tests suggested by a health care provider.

**Conclusion:**

It is imperative that practices and policies are developed and implemented to increase HIV awareness and screening in the older adult population. Increased health care provider awareness of the importance of HIV screening, especially for those 65 and older, is critical. Health policies and clinical guidelines should be revised to promote and support screening of all adults.

## Introduction

Human Immunodeficiency Virus (HIV) has been a major medical and public health challenge over the past three decades. The HIV pandemic has been complicated by the disease’s lack of a cure and its persistent spread, especially in poverty stricken populations and regions of the world. With the advent of newer anti-retroviral drugs, the severity of the disease has been reduced and mortality and morbidity due to opportunistic infections are better controlled in many parts of the world. HIV is now considered a treatable chronic condition, permitting many of those infected to live into old age [1], [2]. Access, affordability, and acceptability remain as continuing barriers to treatment.

HIV/AIDS has not spared any age group, including the elderly. Despite common misperceptions, risky sexual behavior is not limited to adolescents and young adults [3]. While these demographic groups should remain a focus of sexual health programs, the importance of targeting and screening older adults should not be overlooked. It is estimated that by the year 2015, 50% of the HIV-infected individuals in the United States will be 50 years of age and older [4]. Current Centers for Disease Control and Prevention (CDC) guidelines recommend “opt-out” HIV screening for individuals ages 13–64 years in all health-care settings [5], [6]. These guidelines, established in 2006, recommend that patients be notified that testing will be performed, but be given the option to decline or defer testing [5].

In the United States, incidence rates for HIV among persons ages 13 years and older were fairly stable between 2006 and 2009. Annual rates during that period were 48,600 (95% CI: 42,400–54,700) in 2006, 56,000 (95% CI: 49,100–62,900) in 2007, 47,800 (95% CI: 41,800–53,800) in 2008 and 48,100 (95% CI: 42,200–54,000) in 2009 [7]. Minority groups, particularly Blacks and Hispanics, were disproportionately affected. Incidence rates for HIV in persons ages 50 and older are twelve times higher in Blacks and five times higher in Hispanics than in Whites [8]. There is a similar trend in younger age groups. African Americans account for 55% of all HIV infections among those ages 13–24 [9], with young Black men having HIV infection rates that are seven times higher than those for young White men, and three times higher than those for young Hispanic men [10]. Male to male sexual contact remains the highest mode of transmission, followed by injection drug use [11]. Globally, there has been a reduction in the number of individuals newly-infected with HIV. In 2007, there were 2.7 million new HIV cases, a 10% decrease from the previous six years [12]. This decrease is felt to be due to the development of newer, more effective treatments as well as improved screening and prevention strategies.

### Aging in America

There has been a dramatic increase in the number of elderly people in the United States. Life expectancy has increased from approximately 47 years in 1900 to approximately 77 years today [13]. With continued advances in medical care, there may be further increases in longevity over time. While 13% of the population is currently 65 years and older, it is estimated that this figure will be as high as 20% of the population by 2030 [13].

In spite of the fact that there is an increasing number of sexually active older adults, including an increased number living with HIV, studies have shown that older individuals are less likely to be routinely screened or evaluated for HIV infection [14]. Older adults with HIV also often present with symptoms that mimic other diseases, limiting health providers’ level of suspicion for HIV. As a result, they often present with more advanced disease than younger individuals [11], and are more likely to progress to Acquired Immunodeficiency Syndrome (AIDS) [2]. The purpose of this study is to assess the occurrence and barriers to HIV screening in older adults, and to evaluate the value of expanding routine HIV screening to this population.

## Methods

### Ethics Statement

N/A.

### Data Handling

Data obtained from merging portions of the sample adult and person files of the 2009 National Health Interview Survey (NHIS) were used for this study. These data consist of individuals ages 18 and older, but for the purpose of this study, only the 50+ subset (the largest single subgroup at 44.6%) was analyzed. To make for a more meaningful analysis, some categories, like no response, were re-categorized as missing. It was determined that excluding these categories would not change the overall trend of the results. Categories that were small in number were merged and classified as ‘others’ to make for a more useful, descriptive data presentation. The NHIS data collection is achieved through a complex sample design involving stratification, clustering, and multistage sampling with a nonzero probability of selection for each person. Final sampling weights allow estimates from the NHIS to be generalized to the adult civilian population of the United States. To maintain the original sampling design and structure of the survey, subpopulation analyses of the HIV/AIDS, STD, and some TB components of the data were executed, using a complex analysis module.

### Data Analysis and Presentation

Data were analyzed using the complex analysis module in IBM® SPSS® version 20.0 for Windows®. Univariate analysis was performed and results presented using frequency tables with percentages. This analysis classified respondents using socio-demographic and lifestyle variables (Table 1), and knowledge, attitude and practice of HIV testing (Table 2). Bi-variate analysis using Chi-square test, determined the proportional distribution of respondents who had been screened for HIV at least once based on socio-demographic variables and knowledge of an HIV-related disease (Table 3). A multiple logistic regression model was used to determine the odds of ever being screened for HIV (Table 4) and planning to test for HIV within 12 months post-survey (Table 5), for participants engaged in certain high risk behaviors.

**Table 1:** Distribution of Respondents by Socio-Demographic Characteristics.

Variables	Actual frequency *n* (%)	Weighted frequency (*n*)	Percentage(%)
**Sex**			
Male	5 961 (48.2)	43 831 558	46.4
Female	6 405 (51.8)	50 734 479	53.6
**Race by descent**			
White	9 342 (75.5)	71 273 935	75.4
Black	2 033 (16.4)	15 639 056	15.5
Asian	834 (6.7)	6 197 110	6.6
All other races	157 (1.3)	1 455 936	1.5
**Race by ethnicity**			
Hispanic	2 984 (24.1)	24 464 651	25.9
Non-Hispanic white	6 599 (53.4)	49 058 325	51.9
Non-Hispanic black	1 884 (15.2)	14 409 874	15.2
Non-Hispanic Asian	793 (6.4)	5 712 736	6.0
Non-Hispanic others	106 (0.9)	920 451	1.0
**Marital status**			
Married – spouse in household	5 771 (46.7)	57 832 742	61.3
Married – spouse not in household	183 (1.5)	937 683	1.0
Widowed	2 412 (19.5)	12 903 691	13.7
Divorced	2 213 (17.9)	12 130 355	12.8
Separated	380 (3.1)	1 843 869	2.0
Never married	1 059 (8.6)	5 623 182	6.0
Living with partner	318 (2.6)	3 141 401	3.3
**Alcohol drinking status**			
Lifetime abstainer	2 758 (22.3)	18 834 139	19.9
Former infrequent	1 649 (13.3)	12 437 309	13.2
Former regular	1 196 (9.7)	8 319 930	8.8
Current infrequent	1 601 (12.9)	12 534 964	13.3
Current light	2 745 (22.2)	22 570 315	23.9
Current moderate	1 635 (13.2)	13 808 177	14.6
Current heavier	535 (4.3)	4 384 098	4.6
Others	247 (2.0)	1 677 105	1.8
**Reported Health Status**			
Excellent	4 386 (35.5)	33 456 232	35.4
Very good	3 637 (29.4)	28 817 231	30.5
Good	3 108 (25.1)	23 202 798	24.5
Fair	929 (7.5)	6 688 462	7.1
Poor	247 (2.0)	2 268 326	2.4
**Health insurance coverage status**			
Not covered	2 030 (17.1)	16 867 219	18.0
Covered	10 248 (82.9)	76 861 153	82.0
**Type of Medicare coverage**			
Part A – Hospital only	83 (5.4)	524 640	4.7
Part B – Medical only	26 (1.7)	124 849	1.1
Both parts A and B	1 383 (89.6)	10 068 547	90.4
Other	52 (3.4)	356 979	3.2
**Have spent 24+ hrs in the street, shelter or jail/prison**			
Yes	425 (8.4)	4 205 657	4.5
No	4 756 (91.8)	88 846 219	95.5

**Table 2:** Distribution of respondents by their attitude and practice of HIV testing and knowledge of HIV-related disease.

Variables	Actualfrequency *n* (%)	Weightedfrequency (*n*)	Percentage(%)
**Ever been tested for HIV**			
Yes	3 158 (26.8)	22 919 469	25.4
No	8 640 (73.2)	67 377 928	74.6
**Reason for not testing for HIV**			
I am unlikely to have been exposed	6 691 (77.4)	53 248 539	79.5
No particular reason	1 753 (20.3)	12 696 464	19.0
Others	196 (2.3)	1 046 675	1.5
**Time since last HIV test**			
<1 years	63 (5.9)	456 948	5.9
1–2 years	64 (6.0)	480 876	6.2
2–5 years	198 (18.4)	1 385 968	17.8
>5 years	749 (69.7)	5 461 590	70.1
**Main reason for getting HIV test**			
Possible exposure	444 (14.1)	2 508 861	11.0
I just wanted to know	809 (25.7)	5 002 394	21.9
Part of routine check-up	1 178 (37.5)	8 573 763	37.6
Others	711 (22.6)	6 744 678	29.5
**Who suggested the HIV test**			
Healthcare provider	97 (52.7)	642 654	52.7
Sex partner	30 (16.3)	208 960	16.3
Family member	28 (15.2)	204 773	15.2
Other	29 (15.8)	162 355	15.8
**You gave first and last names during test**			
Yes	2 289 (94.0)	20 653 185	93.0
No	146 (6.0)	1 562 963	7.0
**Will be getting an HIV test in the next 12 months**			
Yes	506 (4.2)	3 208 380	3.5
No	11 504 (95.8)	88 922 729	96.5
**Your chances of getting HIV**			
Already have it	50 (0.4)	346 173	0.4
Medium	105 (0.9)	763 703	0.8
Low	1789 (14.8)	13 587 686	14.7
None	10 126 (83.9)	77 772 404	84.1
**Ever heard of tuberculosis (TB)**			
Yes	11 130 (91.6)	86 023 744	91.6
No	1 015 (8.4)	6 920 182	8.4
**Personally know someone with TB**			
Yes	3 304 (29.9)	24 977 059	29.9
No	7 758 (70.1)	60 573 969	70.1
**Knowledge about TB**			
Some or a lot	3 902 (35.2)	30 389 985	35.2
A little	4 829 (45.6)	38 151 659	45.6
Nothing	2 350 (21.2)	17 164 770	21.2

## Results

There was an un-weighted total of 12,366 respondents that were ages 50 years and older. They were almost equally divided into males (46.4%) and females (53.6%). As shown on Table 1, just over 3/4 (75.4%) of respondents were White, with more than half (51.9%) of respondents self-identified as non-Hispanic White. Hispanics were the second largest (25.9%) racial/ethnic demographic group. About 2/3 of the participants were married, with a spouse living in the same household (61.3%), those that were widowed or divorced made up the next largest groups at 13.7% and 12.8% respectively, and about 6.0% stated they had never been married. Lifetime abstainers from alcohol made up about 19.9% of respondents. About 13.2% of respondents had a history of alcohol consumption, while others reported currently consuming alcohol in some quantity, including about 4.6% who reported they were heavy consumers of alcohol. Over 90% of respondents self-reported good, very good, or excellent health, and 81.3% had some form of health insurance coverage. A vast majority (90.4%) of respondents covered by Medicare reported having both Parts A and B. About 4.5% reported having spent 24+ hours on the street or in jail.

Table 2 shows that only 25.4% of respondents reported ever being tested for HIV. Of those who had been tested, almost 70% reported having tested more than five years prior to survey. Of the respondents who had never been tested for HIV, about 79.5% indicated that they felt exposure to the virus was unlikely. The most common reason for HIV testing was a routine medical check-up (37.6%). A majority of respondents (52.7%) reported that a healthcare provider suggested the test. Worthy of note is that only 3.5% of the total participants reported that they planned to get tested for HIV within 12 months following the survey. About 4/5 (84.1%) of the participants rated their chances of contracting HIV as zero (none), and over 98% rated their chances as low or none. As an indicator of knowledge or exposure to other infectious diseases, about 91.6% of respondents had ever heard of tuberculosis (TB), 29.9% knew someone with TB, and 35.2% reported possessing some, or a lot of knowledge about TB.

### Analytical Statistics

Chi-square tests (Table 3) showed that there is a statistically significant difference in the proportional distribution of respondents who had been screened for HIV based on marital status (p<0.001), with widowed respondents, having the lowest percentage (13.9%) of those who have been tested at least once. Similarly, alcohol consumption is strongly associated with the HIV testing practice of respondents; with lifetime abstainers being the group least likely to have been tested for HIV (p<0.001). Those who had been tested for HIV were more likely to repeat the test within 12 months when compared with those who had never been tested for HIV (78.5% vs. 23.3%, p<0.001). The greater the respondents perceived their chance of contracting HIV, the more likely they were to be tested (p<0.001). Respondents who had spent 24+ hours in jail or on the street were more likely to have been tested for HIV than those who had not (52.7% vs. 24.0%, p<0.001). Participants who reported that they had ever heard about tuberculosis were more likely to have been tested for HIV compared with those who reported haven’t ever heard about tuberculosis (25.7% vs. 21.0%, p<0.001). Similarly, respondent’s knowledge of TB and knowing someone with TB was also associated with higher utilization of HIV testing (Table 3).

**Table 3:** Respondent’s lifestyle, knowledge of HIV-related disease and associations with HIV screening.

Variables	Ever been tested for HIV?	
	Weighted frequency *n* (%)	
	Yes	No
Marital status*		
Married – spouse in household	12 383 364 (22.4)	42 882 668 (77.6)
Married – spouse not in household	339 802 (37.0)	579 342 (63.0)
Widowed	1 684 172 (13.9)	10 397 442 (86.1)
Divorced	4 441 695 (38.2)	7 172 653 (61.8)
Separated	770 804 (43.3)	1 008 817 (56.7)
Never married	1 835 315 (33.6)	3 631 537 (66.4)
Living with partner	1 429 731 (47.1)	1 606 939 (52.9)
Alcohol drinking status*		
Lifetime abstainer	3 368 889 (18.7)	14 670 498 (81.3)
Former infrequent	2 992 203 (25.0)	8 973 196 (75.0)
Former regular	2 424 255 (30.5)	5 529 631 (69.5)
Current infrequent	3 228 098 (26.9)	8 781 477 (56.2)
Current light	5 834 356 (26.8)	15 927 599 (73.2)
Current moderate	3 558 295 (26.8)	9 722 091 (73.2)
Current heavier	1 213 769 (29.0)	2 973 306 (71.0)
Will be getting a HIV test in the next 12 mo*		
Yes	2 484 679 (78.5)	679 609 (21.5)
No	20 100 215 (23.3)	66 193 570 (76.7)
Your chances of getting HIV*		
High/Already have it	198 952 (57.5)	147 221 (42.5)
Medium	471 747 (62.3)	285 076 (37.7)
Low	4 506 788 (34.5)	8 553 333 (65.5)
None	17 607 544 (23.3)	57 836 345 (76.7)
Have spent 24+ hrs in the street, shelter or jail/prison*		
Yes	2 133 276 (52.7)	1 914 367 (47.3)
No	20 677 024 (24.0)	65 323 012 (76.0)
Ever heard of tuberculosis (TB)*		
Yes	21 367 633 (25.7)	61 837 434 (74.3)
No	1 412 151 (21.0)	5 323 779 (79.0)
Personally know someone with TB*		
Yes	6 506 195 (27.1)	17 493 468 (72.9)
No	14 799 260 (25.2)	43 969 589 (74.8)
Knowledge about TB*		
A lot	3 497 943 (41.8)	84 866 527 (58.2)
Some	5 736 693 (27.3)	15 247 358 (72.7)
A little	9 094 019 (24.6)	27 836 830 (75.4)
Nothing	3 0099 960 (18.1)	13 646 991 (81.9)

** P<0.001.

Using a multiple logistic regression model and adjusting for sex, race, Medicare type, health insurance coverage status, and self reported physical health status, the odds of previous HIV screening was determined. As shown in Table 4, when compared with married respondents living in the same household with their spouses, only widowed respondents, were less likely to have had an HIV test (OR = 0.7, CI = 0.6–0.8). Similarly, compared with lifetime abstainers, individuals who had used alcohol or were current users of alcohol were more likely to have had an HIV test. Participants who had heard about TB had a higher likelihood of HIV screening at least once prior to the survey, compared with those who had never heard about TB (OR = 1.30, CI = 1.03–1.65). Respondents who reported that they had never lived on the street or been in jail for 24+ hours were less likely to have been tested for HIV (OR = 0.3, CI = 0.2–0.3). Compared with those with a lot of knowledge about TB, having some (OR = 0.5, CI = 0.4–0.6), little (OR = 0.5, CI = 0.4–0.5) or no (OR = 0.3, CI = 0.3–0.4) knowledge of TB were all associated with a lower likelihood of being tested for HIV in this study sample population.

**Table 4:** Results of multiple logistic regression models for ever been tested for HIV adjusted for sex, race, Medicare type, health insurance coverage status, and physical health status.

Variables	Odds ratio (95% CI)
**Marital status**	
Married – spouse in household	1.0 (Reference)
Married – spouse not in household	2.1 (1.4–3.3)*
Widowed	0.7 (0.6–0.8)*
Divorced	2.0 (1.7–2.3)*
Separated	2.6 (2.0–3.6)*
Never married	1.5 (1.3–1.9)*
Living with partner	3.0 (2.2–4.0)*
**Alcohol drinking status**	
Lifetime abstainer	1.0 (Reference)
Former infrequent	1.3 (1.1–1.6)*
Former regular	1.5 (1.2–1.9)*
Current infrequent	1.3 (1.1–1.6)*
Current light	1.3 (1.1–1.5)*
Current moderate	1.2 (1.0–1.5)
Current heavier	1.3 (1.1–1.9)*
**Have spent 24+ hrs in the street, shelter or jail/prison**	
Yes	1.0(Reference)
No	0.3 (0.2–0.3)*
**Your chances of getting HIV**	
High	1.0 (Reference)
Medium	1.2 (0.5–3.0)
Low	0.4 (0.2–0.8)*
None	0.2 (0.1–0.6)*
**Ever heard of tuberculosis (TB)**	
Yes	1.0 (Reference)
No	1.3 (1.1–1.6)*
**Personally know someone with TB**	
Yes	1.0 (References)
No	1.0 (0.9–1.2)
**Your knowledge about TB**	
A lot	1.0 (Reference)
Some	0.5 (0.4–0.6)*
A little	0.5 (0.4–0.5)*
Nothing	0.3 (0.3–0.4)*

** P<0.001.

As presented in Table 5, respondents who were divorced, separated, or never married were more likely to indicate that they planned to get tested for HIV within 12 months post-survey, compared with those who were married. Respondents who had never been tested for HIV prior to the survey were more likely to report that they would not be getting an HIV test within the next 12 months following the survey. But those who reported that they had not spent at least 24+ hours in jail or on the street were 1.54 times more likely to report they planned to get tested for HIV within 12 months post-survey compared with those who had been incarcerated or homeless. Respondents with some knowledge of TB were less likely to indicate a desire for getting an HIV test within 12 months post-survey, while those with little to no knowledge of TB indicated a modestly higher likelihood of getting tested for HIV within the next 12 months after the survey, but this was not statistically significant. Additionally, alcohol consumption was associated with a slightly lower likelihood of getting tested for HIV within 12 months post survey, although this was not statistically significant except for the group which indicated that they were “former regular” alcohol users (p<0.05). It is salient, however, that factors like health insurance coverage, type of Medicare coverage (in the case of eligible participants), health status, and race, which are traditionally predictive of utilization of preventative health services, were not significant in terms of predicting testing for HIV prior to, or within 12 months after the survey (results not shown in the table).

**Table 5:** Multiple logistic regression model showing the odds of getting an HIV test in the next 12 month adjusted for sex, race, Medicare type, health insurance coverage status, and physical health status.

Variables	Odds ratio (95% CI)
**Marital status**	
Married – spouse in household	1.0(Reference)
Married – spouse not in household	2.0(0.7–5.0)
Widowed	1.6(0.9–2.7)
Divorced	1.6(1.2–2.3)*
Separated	2.6(1.6–4.1)*
Never married	2.7(1.8–4.0)*
Living with partner	0.9(0.5–1.8)
**Alcohol drinking status**	
Lifetime abstainer	1.0(Reference)
Former infrequent	0.7(0.4–1.1)
Former regular	0.6(0.4–1.0)**
Current infrequent	0.7(0.4–1.1)
Current light	0.7(0.4–1.2)
Current moderate	0.7(0.5–1.1)
Current heavier	0.5(0.2–1.1)
**Ever been tested for HIV**	
Yes	1.0(Reference)
No	0.1(0.08–0.12)*
**Have spent 24+ hrs in the street, shelter or jail/prison**	
Yes	1.0(Reference)
No	0.1(0.08–0.12)*
**Your chances of getting HIV**	
High	1.0(Reference)
Medium	0.5(0.2–2.0)
Low	0.2(0.1–0.5)*
None	0.1(0.1–0.4)*
**Ever heard of tuberculosis (TB)**	
Yes	1.0(Reference)
No	1.0(0.6–1.5)
**Personally know someone with TB**	
Yes	1.0(Reference)
No	1.1(0.8–1.5)
**Your knowledge about TB**	
A lot	1.0(Reference)
Some	0.9(0.6–1.5)
A little	1.2(0.8–1.9)
Nothing	1.0(0.6–1.7)

** P<0.001;

* P<0.05.

## Discussion

There has been an increase in high risk sexual behavior in older adults [15], [16]. Studies have identified that many adults ages 50 and older have at least one sexual risk factor for HIV, yet they were 6 times less likely to use condoms during sex and 5 times less likely to be screened for HIV, when compared to adults in their twenties with risk factors for HIV [15], [16]. Other studies have shown that the rates of sexually transmitted infections (STI’s) in older adults more than doubled from 1996 to 2003 [13].

Young adults from the 1960s; the era of the “Sexual Revolution”, a time of increased sexual “freedom” and promiscuity, are now in their 60 s and have maintained many of the risky sexual behaviors that became acceptable at that time [17]. Many of these behaviors do not conform to the stereotype of the sexless older person [3]. The misconception that older people do not engage in risky behaviors, including sexual behaviors that may predispose them to HIV, needs to be discarded [18]. Those who have lost their spouses or are divorced may be resuming sexual relationships, potentially exposing themselves to sexually transmitted infections, including HIV. Erectile dysfunction drugs have also contributed to the number of sexually active older men [2]. Postmenopausal older women, with reduced estrogen levels, and atrophic vaginitis, are also at increased risk for acquiring infection [2]. This population of older adults is less likely to utilize barrier methods to prevent pregnancy or STIs [2], and, as shown by the results discussed above, less likely to be screened for HIV (25.4%).

HIV screening for this population occurs most often during a routine medical exam, but, at very low rates. Perceptions among health care providers that older people are less likely to engage in high risk behavior often preclude them from taking an adequate sexual history and truly assessing their risk [19], [20]. Lack of provider awareness is a critical barrier to HIV screening for this older adult population as indicated by our result showing that only 52.7% of respondents indicated that their health care provider recommended the test and that the remaining 47.8% received recommendations to have testing from other sources (Table 2). In view of the cost-effectiveness of screening among persons who might transmit HIV infection others via sexual behaviors or injection drug use practices, the American College of Physicians (ACP) recommends that physicians routinely encourage HIV testing for all adults up to at least the age of 75 years [21], [22]. This study did not find any statistically significant difference in HIV testing by race; probably due to the sample populations’ equal access to health insurance (about 96.2% were covered by some form of Medicare). Other studies have documented that HIV positive older African Americans reported that age was a major barrier to seeking services and support [23]. This study shows that perceived HIV risk has an effect on HIV screening as 84.1% of the respondents perceived their risk of contracting the virus as zero and 91.4% (not shown in tables) reported that they did not plan to get tested within 12 months following the survey. Individuals who were widowed and those who had not spent any time in jail or on the street were also less likely to have been tested for HIV at the time the survey was administered, but indicated a slightly higher likelihood of obtaining the test within 12 months post survey. Lower screening rates within the elderly population has been attributed to lower perception of risk due to poor knowledge of HIV, HIV transmission, and safer sex practices as well as failure on the part of providers to recommend HIV screening [24]–[26]. This point is well supported by our data which showed that lower perception of HIV risk was associated with a decreased likelihood for getting an HIV test. Current or prior history of alcohol use was associated with a higher odds of getting an HIV test at least once at the time of the survey, although this was also associated with a lower although not statistically significant odds of getting an HIV test within12 months following the survey. Alcohol use behavior might have led to an increased risk perception and thus the need to get tested for those who reported having been tested during the survey. For example, studies show that among younger adults, alcohol consumption is associated with safe-sex practices and thus increased risk perception for HIV exposure [27], [28]. Although we are not sure why the desire to test for HIV was lower among the same group, we hypothesize that a prior negative result might have led to a lower risk perception for these individuals, leading to a decreased perception to test despite the presence of the risk factor; in this case alcohol use or a history of it.

Of note is the strong relationship between marital status and HIV screening among the participants. The reason for this is not immediately apparent to the authors since the survey did not address this. But we hypothesize that a higher risk perception for exposure to HIV among respondents who were married, but not living with their spouse, separated, never married and living with their unmarried partner led to a higher testing rate for HIV than those who were married and living with their spouse [29], [30]. It is also possible that respondents and their spouses perceive that at this age, there is less likelihood of either partner having multiple sexual partners; thus decreased risk for exposure to sexual transmission of HIV when married and living with spouse. This could explain the slightly higher albeit not statistically significant odds of testing for HIV among the other groups in the marital status category (except those living with a partner to which they were not married) compared with the group that were married and living with their spouse. The accuracy of our hypothesis will require a study designed to answer this question and others like it.

Most of the participants who had either spent time on the streets, jail, or who had some knowledge of a disease associated with HIV (in this case, TB) were more likely to have been screened. The higher screening rates for those who had spent time in jail may be due to CDC-recommended routine HIV testing in jails and prisons [31], [32], mandatory HIV testing for inmates in some states and the federal prison system, [33] or court mandated HIV screening. The high screening rates for older adults who had spent time on the streets may be due to public health HIV prevention strategies directed at homeless populations based on the 2006 revised CDC HIV screening recommendations [5]. From the results, knowledge of TB (a disease associated with HIV) increases the likelihood of screening for HIV. This may be a result of increased knowledge of HIV infection among those with related infectious diseases. This suggests that there is a value to promoting HIV awareness and screening in settings where other infectious diseases are being screened and treated. This result supports the findings of other investigators who reported that low knowledge of TB was associated with low knowledge of HIV [34]. Similarly, patients with TB will readily accept HIV testing when it is anonymous and unlinked [35], As Reid et al [36] emphasized in their review, tuberculosis control programs play a very important role in HIV control and prevention via providing HIV education, and opportunities for testing in addition to early and readily available access to medication.

## Limitations

Like any study that utilizes secondary data, this study had some limitations. First, the study depended on a self reported survey which is subject to participant recall bias; as such, information provided cannot be validated. Second, underreporting of sensitive information such as HIV screening and risk factors may affect results. Third, the study utilized already coded NHIS data, which prevented analysis of HIV testing rates by subsets of potential interest such as variations in testing behavior versus each decade increase in age. Fourth, the survey did not include specific questions on sexual practices and sexual orientation and thus it is impossible to access how these might affect HIV testing behavior among this demographic. Finally, the NHIS survey excludes military personnel on active duty and other individuals who live outside households, including persons who are incarcerated, in long-term care institutions, or homeless. Certain persons in these populations might be at greater risk for HIV infection than persons living in households, therefore skewing study results.

### Conclusion

Interventions aimed at improving policies and practices that will increase HIV screening within the older adult population must be encouraged. Efforts should be made to increase knowledge about HIV and the importance of HIV screening among individuals’ ages 50 years and older, especially those 65 and older, a population in the U.S. that is dramatically increasing. Awareness of the importance of HIV screening for this population should also be promoted among health care providers. Health care providers should be proactive in screening and screening guidelines, including the CDC’s screening recommendation for adults, should be revised to include this older demographic. We recommend that the age limit be eliminated, and “opt out” screening advised for all adults. Broadening the screening guidelines will not only enable us to capture this important, largely unscreened age demographic, but also open up opportunities for discussions about HIV, its predisposing factors, and modes of prevention between providers and their older patients.
